# Nutrient Recovery from Digestate of Agricultural Biogas Plants: A Comparative Study of Innovative Biocoal-Based Additives in Laboratory and Full-Scale Experiments

**DOI:** 10.3390/molecules27165289

**Published:** 2022-08-19

**Authors:** Ievgeniia Morozova, Andreas Lemmer

**Affiliations:** State Institute of Agricultural Engineering and Bioenergy, University of Hohenheim, 70599 Stuttgart, Germany

**Keywords:** digestate valorization, biocoal (biochar), phosphorus, ammonium, potassium, screw press, anaerobic digestion, separation, additives

## Abstract

Nutrients can be recovered from the digestate of an agricultural biogas plant in the form of solid fraction and serve as crop fertilizers. Removal of suspended solids with screw press separators is the most commonly used technique for treating digestate from biogas plants. To increase separation efficiency and nutrient transfer to the solid phase during separation, eight biocoal-based additives were investigated, which were based on beech wood and produced by pyrolysis at temperatures of 350 °C and 600 °C. Four of the biocoals were impregnated with CaCl_2_ or MgCl_2_ before pyrolysis. The reaction time between the additives and the digestate varied from 5 min to 2 weeks. The application of MgCl_2_-impregnated biocoal synthesized at 600 °C for 20 h increased the nutrient removal efficiency by 76.33% for ammonium and 47.15% for phosphorus, compared to the control (the untreated digestate).

## 1. Introduction

Phosphate fertilizers such as diammonium phosphate (DAP), are essential for plant growth and are made from phosphate rock. Phosphate rock and DAP prices are expected to increase 1.6-fold and 1.4-fold, respectively, by 2035. The main reason for this is the increasing demand for phosphate and the depleting phosphate rock reserves [1,2,3]. On the other hand, there is a major problem of ammonia leaching, ammonia emissions, and eutrophication due to excess ammonia in regions with high livestock density, where meat processing factories are often located [4,5]. Both issues could be solved by sustainable nutrient recovery from slurry, either by direct treatment or by processing the digestate of biogas plants after anaerobic digestion of the slurry.

Various mechanical and non-mechanical techniques for nutrient recovery from digestate have been studied: sedimentation, centrifugation, drainage, pressure filtration and chemical pretreatment (by precipitation, coagulation and flocculation), use of specific additives (sorbents), among others [6,7,8,9,10]. In full-scale biogas plants, pressure filtration with a screw press is commonly used [11]. This method is costly and energy consuming, and its efficiency needs to be improved [10]. In this study, the efficiency of nutrient binding of biocoal-based additives was investigated when applied before a mechanical separation step (pressure filtration) to improve nutrient recovery in the solid fraction of the digestate. Although the liquid fraction of digestate is a valuable nutrient source [12,13,14,15,16], the focus on the solid fraction in this study can be explained by lower transportation and storage costs for its application as fertilizer for crops cultivation in times of their nutrient demand.

For cultivating crops, an improved nutrients retention and their slow release was confirmed on charcoal-rich soils (i.e. Terra Preta) due to the high cation exchange capacity and high porosity of charcoal [17]. In the same way, the application of biochar to the soils proved to improve plant yields by 15–17%: for arbuscular mycorrhizal fungi, it stimulated root development and increased root colonization; for rice, biomass yields and soil pH were increased; for maize, the stimulated plant growth with the improved root system was observed [17].

Literature sources use different terms, i.e. hydrochar or hydrocoal, biochar or biocoal depending on the origin and production technology [18,19,20,21,22]. The terms “biochar” and “hydrochar” are commonly used when the products are used as fertilizers and made of biomass, agricultural residues, or wastes [19,20,21]. In this study, nutrient recovery from digestate is investigated based on the sorption characteristics of these additives. Therefore, the terms “hydrocoal” and “biocoal” (referring to “hydrochar” and “biochar”) will be used in the following. The main difference between biocoal and hydrocoal lies in their production process: biocoal is a product of a thermochemical process, such as pyrolysis or torrefaction (so-called mild pyrolysis), while hydrocoal is obtained by hydrothermal carbonization (HTC) [18,19,20,22]. The quality and properties of biocoal or hydrocoal are directly related to the process temperature and heating time (residence time of the material in the reactor); the heating rate (for slow pyrolysis and torrefaction), pressure, and moisture content of the material have an additional influence [18,21]. The pyrolysis process is carried out by heating the material in the temperature range between 300 °C and 650 °C in the absence of oxygen [18]; most commonly, biocoal is synthesized under N_2_ flow [23,24,25,26,27]. Torrefaction is achieved by limiting the process temperature to 200–300 °C and the heating time from 30 min to several hours with heating rates of less than 50 °C∙min^−1^ [18,19]. HTC or the so-called wet torrefaction is carried out in the temperature range of 180–260 °C. In HTC, raw material is immersed in water and heated in a closed system under pressure (2–6 MPa) for 5–240 min [18,22].

The recovery of nutrients from municipal or industrial wastewater using biocoal has already been investigated in several studies [23,24,25,26,27,28,29,30,31]. Efficient phosphate recovery from piggery digestate when using biogas residue biocoal was described by Luo and co-authors [32]; however, this method is resource-consuming (due to chemicals application), and the recovered phosphate is available as a magnesium ammonium phosphate mixture. Although the aforementioned studies showed the positive effect of the biocoals they used for nutrient recovery, research into new types of biocoals for commercial application is needed. According to Harikishore and co-authors, the main obstacle for nutrient adsorption by using biocoal is the negatively charged surface of biocoal and the anionic nature of nitrate and phosphate molecules [33].

Impregnation of the feedstock with MgCl_2_ or CaCl_2_ prior to biocoal production has improved the sorption capacity of biocoal [9,10,24,25,34]. The usefulness of MgCl_2_ and CaCl_2_ can be described due to formation of porous crystals of magnesium oxide and calcium oxide during biocoal production, which are valuable adsorbents [34,35]. MgCl_2_-salt is added to enhance phosphorus recovery via magnesium ammonium phosphate (MAP, sturvite) precipitation from wastewater or digestate [9]. Biocoal from Mg-enriched tomato leaves synthesized at 600 °C was studied by Yao and co-authors [24]. Corncob based biocoal enriched in Ca and Mg and pyrolyzed at 300 °C, 450 °C, and 600 °C was analyzed by Fang and co-authors [25]. Additionally, the temperature of the biocoal synthesis also seems to have an effect on nutrient recovery. Fang and co-authors revealed that higher phosphate recovery was achieved when biocoal synthesized at higher process temperatures was used [25]. Mg-modified sugarcane bagasse biocoal pyrolysed at 700 °C was efficient for P-absorption from acid-extract of incinerated sewage sludge ash [34].

In this study, the nutrient binding into the solid fraction after the pretreatment of biogas digestate with the biocoal-based additives and subsequent solid-liquid separation was investigated. For this purpose, digestate treated with the additives and untreated digestate were separated by pressure filtration. Biocoal-based additives of different origins synthesized at different process temperatures and both impregnated and not impregnated with CaCl_2_ or MgCl_2_ were investigated. Additionally, the effect of different reaction times, i.e., the period of digestate pretreatment with the additives, was analyzed. The research results were compared with the control variant, corresponding to the untreated digestate.

## 2. Materials and Methods

### 2.1. Experiment Overview

In this study, the experiments were conducted both in full-scale and under laboratory conditions. First, the separation experiments were conducted in the full-scale research biogas plant “Unterer Lindenhof” described in some published papers [36,37,38]. In the separation experiments, pressure filtration (400 mbar inlet pressure) was performed using a screw press. The aim of the full-scale separation experiments was to identify benchmark values for a comparable control in the laboratory. These benchmark values were used to adjust the pressure filtration in the laboratory to allow comparable results. During the full-scale separation, different screw press settings were used to evaluate minimum and maximum solid-liquid and nutrient separation efficiency. The laboratory-scale experiments comprised both the separation of untreated digestate and the separation experiments with the digestate pretreated with additives. First, the separation experiments with untreated digestate were performed and the optimum separation settings were determined. The optimum was selected based on the research results, which correspond to the mean benchmark values obtained in full-scale. The optimum separation settings were then applied to separate the digestate pretreated with additives. To evaluate the effect of the additives on nutrient removal efficiency, they were exposed to the digestate prior to separation at a defined ratio and for a specific reaction time.

#### 2.1.1. Mechanical Separation Step in the Full-Scale Application

**Figure 1:**
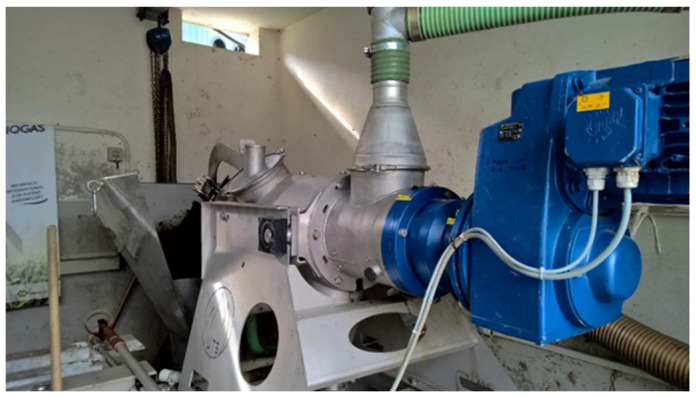
A filter screw press used for the digestate separation at the full-scale research biogas plant of University Hohenheim “Unterer Lindenhof”.

Full-scale mechanical separation experiments were conducted at the research biogas plant of University Hohenheim “Unterer Lindenhof” located at “Eningen unter Achalm”, Baden-Württemberg, Germany. A filter screw press FSP-A 20150518 (UTS Products GmbH, Dorfen, Germany) was used for digestate separation (see Figure 1).

The anaerobic digestion process at “Unterer Lindenhof” is organized in two steps. The main fermentation process takes place in the anaerobic reactors #1 and #2. In a se-condary reactor the residual fermentation takes place. The digestate from the secondary reactor was selected for the separation experiments. The content of the reactor was mixed for five minutes prior to the separation step to achieve uniform distribution of total solids.

Three configurations of the screw press were tested. The digestate and collected separated fractions were frozen and stored at −20 °C before analysis in the laboratory.

#### 2.1.2. Mechanical Separation Step in Laboratory Conditions

**Figure 2:**
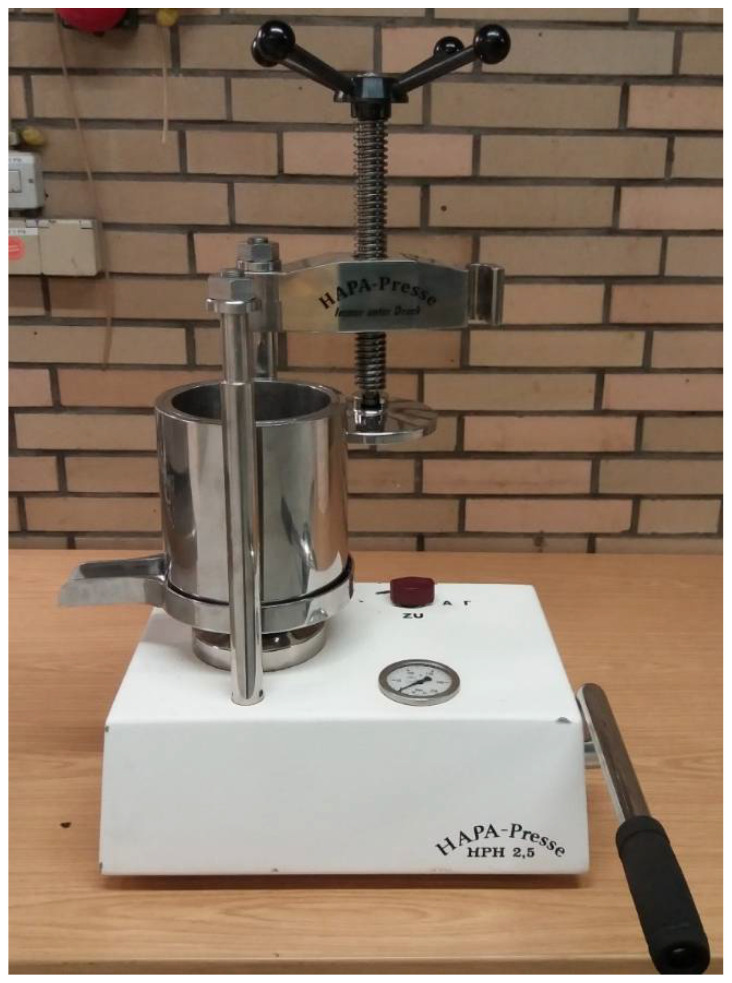
A hydraulic tincture press used for the digestate separation experiments in the laboratory.

The separation experiments in the laboratory were conducted using a hydraulic tincture press (HAPA HPH 2.5 l, Achern, Germany), see Figure 2. The operation mechanism of the press is a combination of spindle and hydraulic system. The following operating conditions were tested in advance to find the optimal separation settings: (1) Different amounts of digestate in a range between 170 g and 1000 g. (2) Different pressure modes: atmospheric pressure, 25 bar, 50 bar, 75 bar, 80 bar, 100 bar and 125 bar. (3) Different duration of pressure application: 5 s, 60 s, 300 s. The settings resulting in the highest agreement with the benchmark values from the full-scale separation were selected for further experiments. In our case, the selected separation regime was 300 g sample pressed under 100 bar for 60 s.

#### 2.1.3. Pretreatment with Additives before the Mechanical Separation Step

Different types of biocoal-based additives were analyzed in this study. The biocoals produced within this research are specified in Section 2.2. In addition, a commercial biocoal was analyzed and tested either alone or in combination with MgCl_2_-salt. The amounts of additives added to the digestate were calculated as described in Section 2.4. Different reaction conditions were analyzed, such as mixing of digestate with additives over diffe- rent time intervals and storage with short mixing times (see Section 2.4). After pretreatment of the digestate with additives, it was immediately used for the mechanical separation step under the operating conditions assumed to be optimal as described in Section 2.1.2.

### 2.2. Production of Biocoals

Six biocoal variants were produced from beech wood by pyrolysis. Two biocoal variants were untreated beech wood synthesized at 350 °C (Biocoal V1) and 600 °C (Biocoal V4), respectively. Further, two biocoal variants were synthesized at 350 °C (Biocoal V2) and 600 °C (Biocoal V5), respectively, after impregnation with a CaCl_2_-salt solution with a concentration of 5.69 mol/L. The last two biocoal variants were based on beech wood impregnated with MgCl_2_-salt solution (5.69 mol/L) with the subsequent pyrolysis at either 350 °C (Biocoal V3) or 600 °C (Biocoal V6).

The wood was impregnated manually in twelve 30-l barrels for three weeks at the University of Hohenheim. Mixing was conducted by rolling each barrel for 30 min daily. Excess liquid was removed by drying the impregnated wood at 60 °C for 17–18 days in a drying chamber (Robert Hildebrand Maschinenbau GmbH, Oberboihingen, Germany) to a constant weight.

Both impregnated and unimpregnated wood was delivered to the Clausthal Research Center for Environmental Technologies (CUTEC), where it was pyrolyzed in a rotary kiln [39,40]. For the synthesis of biocoal at either 350 °C or 600 °C, the walls of the pyrolyzer were heated accordingly for 8–12 h. The average retention time of the solid material in the pyrolyzer was 45–60 min. The pyrolyzer was filled to about 10–20% with wood. The synthesis of biocoal was carried out under N_2_ flow, and it took about 18 h to produce a biocoal variant.

### 2.3. Analysis of Biocoals

The biocoals were analyzed for their specific water uptake (see Section 2.3.1), elemental composition, bulk density, and ash content (see Section 2.3.2).

#### 2.3.1. Specific Water Uptake Analysis

For the analysis of specific water uptake, 250-mL bottles were filled with 125 mL bio-coal and 125 mL deionized water at 20 °C. Specific water uptake for the biocoals was measured in triplicates after 1 h, 1 day, and 1 week. After the exposure period, the samples were filtered with a sieve (100 μm mesh size) to remove excess water. The biocoal was weighed before ($m_{start}$) and after ($m_{end}$) exposure, and specific water uptake was calculated according to Equation (1):

(1) $$Specific\;water\;uptake=\left(m_{end}\cdot {\left(m_{start}\right)}^{-1}-1\right)\cdot 100\%$$

#### 2.3.2. Analysis of Biocoals

The proportion of C, H, and N in the biocoals was measured using a Euro3000 EA CHNSO elemental analyzer (HEKAtech GmbH with Callidus software interface version 5.1, Wegberg, Germany). Analyses were conducted after spontaneous combustion at 1000 °C followed by chromatographic separation.

Ca and Mg contents of the biocoals were measured using inductively coupled plasma mass spectrometry (ICP-MS) with a NexION 2000 (PerkinElmer, Rodgau, Germany) after microwave digestion (Discover SP-D, CEM GmbH, Kamp-Lintfort, Germany).

For measuring bulk density, a bulk density cylinder was used. The dry matter (DM) content as DM relative to fresh matter (g∙kg_FM_^−1^) and the organic dry matter (oDM) content as oDM relative to DM of the samples (in g∙kg_DM_^−1^) were determined by differential weighing before and after drying at 105 °C for 24 h and after subsequent ashing at 550 °C for 8 h, respectively, using standard methods [41].

### 2.4. Experimental Design

The digestate was pretreated with additives in 2 L bottles. In total, seven variants of biocoal and six reaction times were tested. Reaction time refers to the period between the addition of biocoal to the digestate and solid-liquid separation. Among the six reaction times, four reaction times corresponded to periods of continuous mixing at 300 rpm on an orbital shaker (IKA KS260, Staufen, Germany) at 20 ± 2 °C for either 5 min, 1 h, 3 h or 20 h. Two further reaction times were established, when the digestate and the additives were first mixed for 5 min and then stored at 20 ± 2 °C for 1 or 2 weeks, respectively, with additional 5 min mixing intervals every second to third day.

An overview of the experimental design is shown in Table 1. The commercial biocoal was originally produced for the production of TerraPreta. It was added in the amount recommended by the manufacturer: 6 L of the biocoal per 1 m^3^, which was equivalent to 8.33 g biocoal per kg digestate. The amounts of the CaCl_2_-biocoals, MgCl_2_-biocoals and MgCl_2_-salt added to the digestate were calculated for the equimolar concentrations of Ca or Mg in the biocoals and of P in the digestate, allowing for the precipitation of magnesium-ammonium-phosphate possible. The amounts of the other biocoal variants were calculated under consideration of their individual bulk densities. For the pretreatment with the additives, 2 L bottles filled with 1.2 kg of the digestate were used, and thus, the presented amounts of additives applied corresponded to 1.2 kg of the digestate (see Table 1).

### 2.5. Analytical Methods

The DM content of the collected samples was determined in the same way as for the biocoals, which is described in Section 2.3.2. NH_4_^+^ concentrations in the digestate and the separated fractions were determined using the Gerhardt Vapodest 50 s automatic distillation system (Germany). In the further text, NH_4_ is written, which means the ammonium ion NH_4_^+^. Potassium was determined using flame atomic absorption spectroscopy (AAS, Eppendorf, ELEX 6361, Hamburg, Germany) operated with acetylene gas. For the determination of phosphorus, a cuvette test and a spectrophotometer UV-VIS 1240 (Shimadzu, Kyoto, Japan) were used. All analyses were carried out according to standard methods [41].

### 2.6. Calculation of Removal Efficiency

The influence of the different additives on the removal of nutrients during separation was evaluated on the basis of the parameter “removal efficiency”, which describes the fraction of nutrients of the initial substrate that was contained in the solid phase after se-paration. The removal efficiency for different nutrients was calculated according to Equation (2):

(2) $$\mathrm{Removal}\;\mathrm{efficiency}=Quantity\;in\;solid\;fraction\cdot Quantity\;in\;digestate^{-1}\cdot 100\%$$

### 2.7. Calculation of Sorption Capacity

The sorption capacity of P or PO_4_^3−^ ($q_{e}$, mg∙g_biocoal_^−1^) was calculated as described by Li and co-authors [26] and defined by Equation (3):

(3) $$q_{e}=M\cdot C_{s.f.}\cdot m^{-1}$$

where M is the quantity of digestate used for the pretreatment with the biocoals (g); $C_{s.f.}$ is the concentration of P or PO_4_^3−^ in the solid fraction after separation (mg∙g^−1^), m is the quantity of the biocoal supplied to M for its pretreatment under the specific reaction time according to the experimental design with the following separation step (g).

### 2.8. Statistical Analysis

Microsoft EXCEL 2016, R and RStudio (version 1.1.463) and SAS 9.4 were used for data processing and visualization. In the statistical analysis, the post hoc Tukey HSD test and the generalized linear model function were applied as described by some authors [42,43].

## 3. Results and Discussion

### 3.1. Results of the Mechanical Separation Step without Pretreatment: Comparison of Full-Scale and Laboratory Experiments

**Figure 3:**
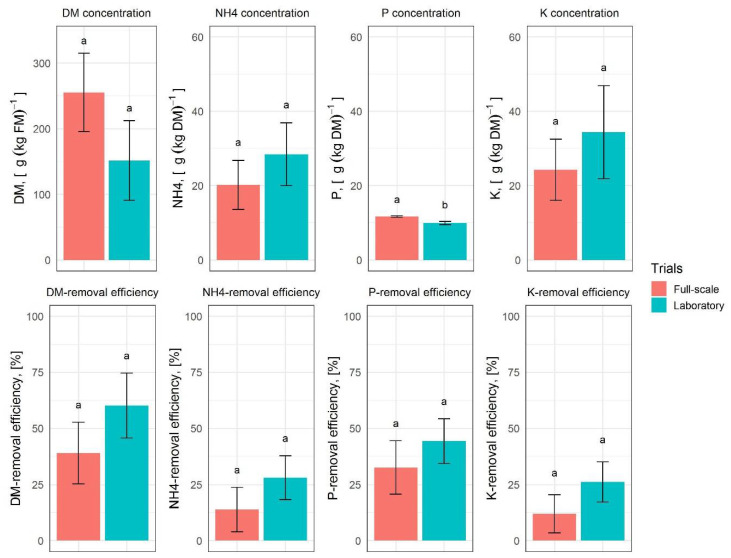
Results of the separation trials in the full-scale and laboratory systems. The upper diagrams show the concentration of nutrients in the full-scale and laboratory. The lower diagrams show the removal efficiency. Histograms are charted based on the mean values; error bars indicate the variability between the three replications. Lower case letters indicate significant differences accor-ding to the results of the Tukey test.

The digestate for the separation experiments was taken from the secondary reactor of the research biogas plant “Unterer Linderhof”. During the experimental period, the reactors were fed with the following substrates: liquid manure, maize silage, grass silage, solid manure, horse dung, WPS (whole plant silage), sugar beet, cereals and water. The operating conditions during the experimental period and the properties of the digestate are shown in Table 2:

For the full-scale system, the FM removal efficiency in the solid fraction varied from 7.36% to 23.92%, depending on the different set-ups of the screw press. The screw press set-up with the highest FM removal efficiency yielded the highest removal efficiency for all parameters analyzed and was selected as the baseline setting that provided the benchmark results for the laboratory trials as shown in Figure 3.

The results of the laboratory separation experiments are comparable to those obtained in full-scale and no significant differences were found between both systems, except for P concentration. Although the absolute difference between P concentrations in laboratory and full-scale with 9.92 ± 0.48 g∙kg_DM_^−1^ and 11.67 ± 0.23 g∙kg_DM_^−1^, respectively, was very small, it was statistically significant due to the very small variance of the mea- sured values.

The preliminary studies demonstrated that similar separation efficiencies can be achieved in the laboratory with the tincture press as in practice with the screw press. Mechanical separation in the laboratory for the untreated digestate is the control variant to evaluate the effect of the tested additives in further investigations.

### 3.2. Results of the Laboratory Analysis of the Biocoals

The biocoals produced for this study were first analyzed for their chemical and phy-sical parameters. The following table shows the results of elemental analysis of the biocoals without impregnation depending on pyrolysis temperature (Table 3).

Higher process temperature during pyrolysis resulted in a higher carbonization, while H and N contents were in the same range for biocoal variants produced under both synthesis temperatures. The C contents of Biocoals V1 and Biocoal V4 were in the same range as the biocoal-based fertilizer described by Rasse and co-authors, with the C contents equal to 75 ± 15% [17].

The results of the supplementary analyses, such as bulk density, DM and oDM contents, Ca and Mg contents for all biocoals used are shown in Table 4.

Impregnation of wood with CaCl_2_ or MgCl_2_ prior to pyrolysis resulted in a higher density of the charcoal produced due to the increased mineral content. Higher synthesis temperatures resulted in the smaller biocoal particle size. Pretreatment with CaCl_2_ increased the Ca content of the biocoal by a factor of 45 (Biocoal V2) and 21 (Biocoal V5), respectively, compared to the untreated biocoal variants at the same synthesis temperatures. Pretreatment with MgCl_2_ increased the Mg concentration by a factor of 66 (Biocoal V3) and 53 (Biocoal V6), respectively, compared to the untreated biocoal variants at the same synthesis temperatures.

### 3.3. Water Uptake of the Biocoals

**Figure 4:**
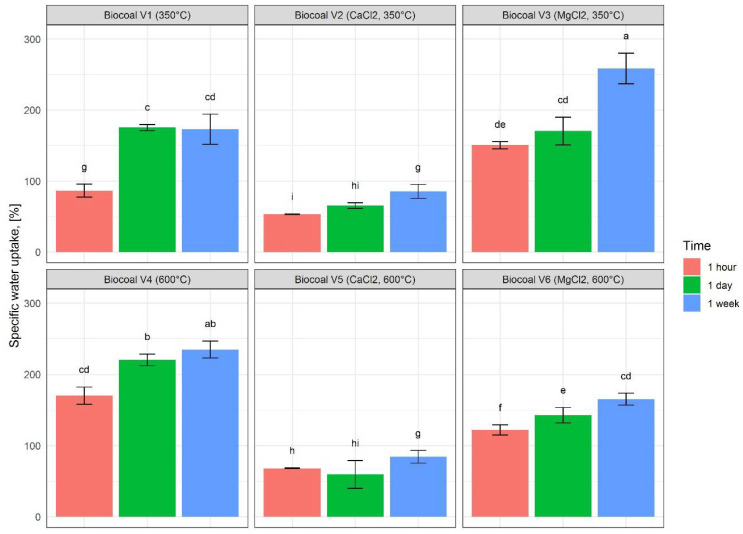
Specific water uptake for the six produced variants of biocoal under different exposure times (one hour, one day and one week). Histograms are charted based on the mean values; error bars indicate the variability between the three replications. Lower case letters indicate significant differences between all the experiments according to Tukey test.

Specific water uptake is an important parameter for the application of biocoal as a soil conditioner. The results on specific water uptake for the six produced variants of biocoal under different exposure times (one hour, one day and one week) are shown in Figure 4.

With increasing exposure time, the specific water uptake of biocoals increased (see Figure 4). The highest specific water uptake of 250.49% was measured for Biocoal V3 after one week exposure. Pretreatment with CaCl_2_ resulted in a lower specific water uptake compared to the other biocoal variants.

### 3.4. Results on Nutrients Recovery after Pretreatment

In some variants of the laboratory tests, no sufficient dewatering of the digestate could be achieved with the tincture press. Therefore, all variants with a fresh mass fraction of the solid phase after separation above 50% were excluded from further investigations. Thus, all experimental results obtained for the reaction time of one and two weeks and for Biocoal V2 were omitted. Digestate, solid- and liquid fractions were analyzed on their nutrient contents. The results on nutrient contents for the liquid fraction were considered for calculating mass balances and validating the nutrient recovery in the solid fraction; however, due to the main focus on the solid fraction, the results for the liquid fraction are not described in this study.

#### 3.4.1. FM-Removal, DM Concentration and DM-Removal Efficiency

**Figure 5:**
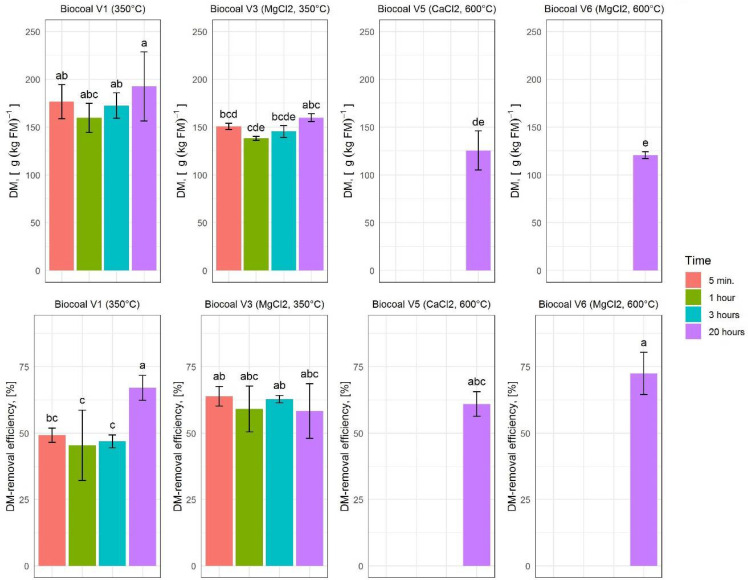
Dry matter (DM) concentration and DM-removal efficiency for the different variants of added biocoal. The upper diagrams show the DM concentration. The lower diagrams show the removal efficiency. Histograms are charted based on the mean values; error bars indicate the varia-bility between the three replications. Lower case letters indicate significant differences according to Tukey test.

First, the effect of the additives on DM concentration of the solid phase after separation and on the degree of separation of the solids was investigated. The results on DM concentration and DM-removal efficiency for the ten tested variants are given in Figure 5.

The highest DM concentration in the solid phase for the four tested reaction times were measured for Biocoal V1 with 175.39 ± 21.20 g∙kg_FM_^−1^ (see Figure 5). The highest DM-removal efficiency was found for the reaction time of 20 h. However, the variant of biocoal also affected the FM and DM-removal efficiencies, with Biocoal V6 having the highest DM-removal efficiency of 78.06%.

#### 3.4.2. NH_4_ Concentration and NH_4_-Removal Efficiency

**Figure 6:**
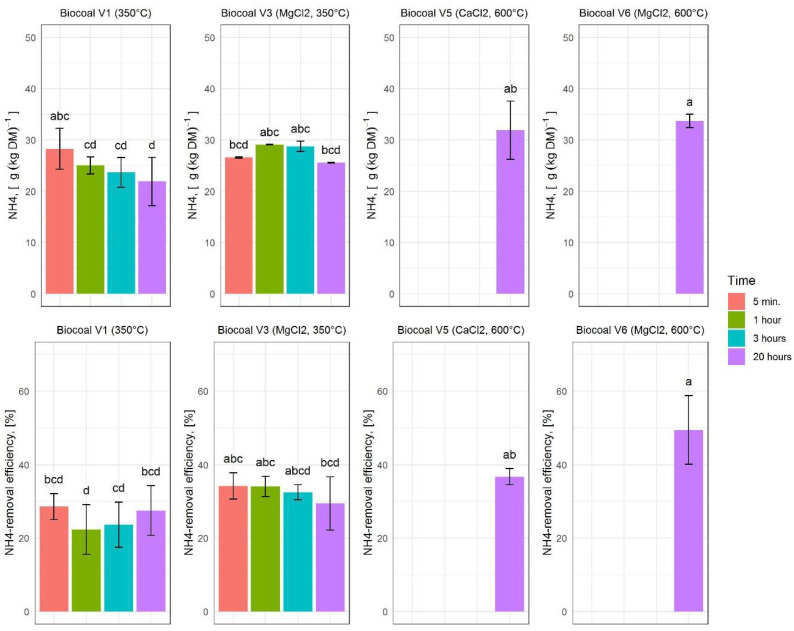
Ammonium (NH_4_) concentration and NH_4_-removal efficiency for the different variants of added biocoal. The upper diagrams show the NH_4_ concentration. The lower diagrams show the removal efficiency. Histograms are charted based on the mean values; error bars indicate the variability between the three replications. Lower case letters indicate significant differences according to Tukey test.

Additives are used to increase the NH_4_-concentration in the solid phase and thus, improve the removal efficiency. The highest NH_4_ concentration of 34.63 g∙kg_DM_^−1^ was measured in the solid fraction after pretreatment with Biocoal V6 at the reaction time of 20 h (see Figure 6). The biocoal variants synthesized at higher temperatures had a higher NH_4_-removal efficiency in the solid fraction. Among all tested variants, the highest NH_4_-removal efficiency of 56.04% was measured for Biocoal V6 after pretreatment for 20 h.

The NH_4_ removal efficiency observed in this study is up to 1.8 times higher than reported by Kocatürk-Schumacher [28] and more than 3 times higher than reported by Takaya and co-authors [29]. Kocatürk-Schumacher applied biocoal from holm oak at a synthesis temperature of 650 °C and NH_4_ removal efficiencies between 10% and 32% were observed [28]. Further, application of different types of hydrocoals and biocoals synthesized in the temperature range between 250 and 650 °C led to a removal efficiency of NH_4_-N between 9% and 17% [29].

#### 3.4.3. P Concentration and P-Removal Efficiency

**Figure 7:**
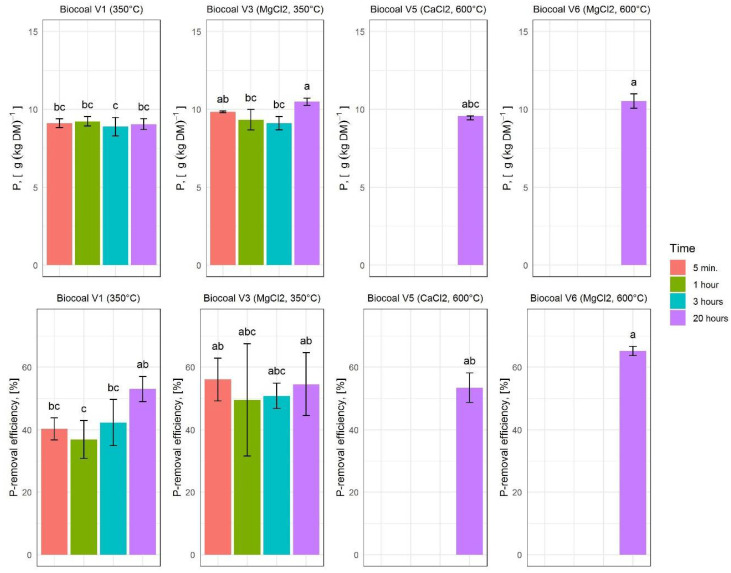
Phosphorus (P) concentration and P-removal efficiency for the different variants of added biocoal. The upper diagrams show the P concentration. The lower diagrams show the removal efficiency. Histograms are charted based on the mean values; error bars indicate the variability between the three replications. Lower case letters indicate significant differences according to Tukey test.

According to the control experiments, the amount of P to be removed in the solid fraction is 38.41 ± 11.70%, based on the total amount of P in the digestate. The P-removal efficiency needs to be significantly improved for the commercial use of the P-rich solid fraction. Thus, by testing the additives, the P concentrations and P-removal efficiency were determined as shown in Figure 7.

The highest P concentrations were measured in the separated samples after the digestate pretreatment with the Mg-rich biocoals.

Application of biocoals loaded with CaCl_2_ or MgCl_2_ led to higher P-removal efficiencies than application of unloaded Biocoal V1. Mean P-removal efficiency for Biocoal V3 and Biocoal V5 was in the same range. The highest P-removal efficiency of 65.18 ± 1.48% was found after application of Biocoal V6 for 20 h.

To compare the results obtained in this study with those from literature, the sorption capacities for P and PO_4_^3−^ were calculated. Neither the wood impregnation with MgCl_2_ or CaCl_2_ nor different synthesis temperatures improved the sorption capacities of P and PO_4_^3−^ compared to Biocoal V1. Nevertheless, biocoals impregnated with MgCl_2_-resulted in higher sorption capacities than biocoals impregnated with CaCl_2_. The sorption capacities for all biocoal variants tested were much higher than those reported in literature [23,25,26,27,28,29,44].

#### 3.4.4. K-Concentration and K-Removal Efficiency

**Figure 8:**
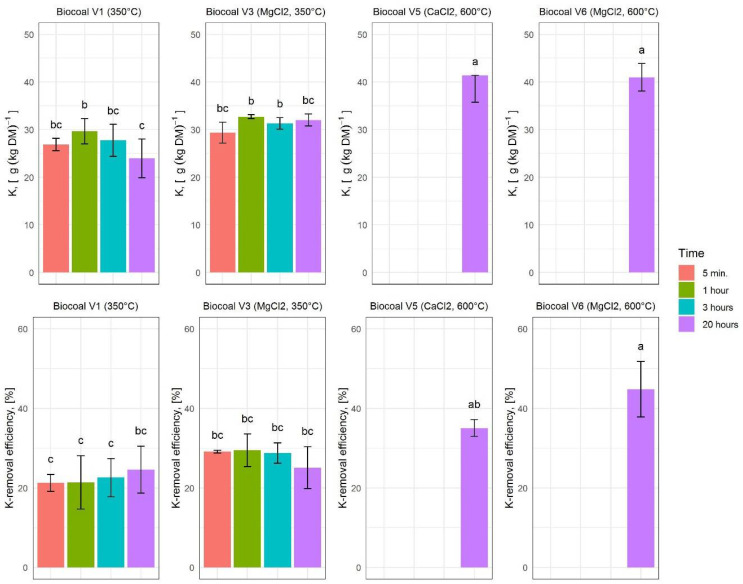
Potassium (K) concentration and K-removal efficiency for the different variants of added biocoal. The upper diagrams show the K concentration. The lower diagrams show the removal efficiency. Histograms are charted based on the mean values; error bars indicate the variability between the three replications. Lower case letters indicate significant differences according to Tukey test.

As digestate is rich in K, which is an essential crop nutrient, improved K-removal into the solid fraction is of relevance for plant nutrition. The K concentration and K-removal efficiency results for the analyzed variants are given in Figure 8 and described below.

### 3.5. Comparison of the Control Variant with Those Pretreated with Additives with the Best Performance

**Figure 9:**
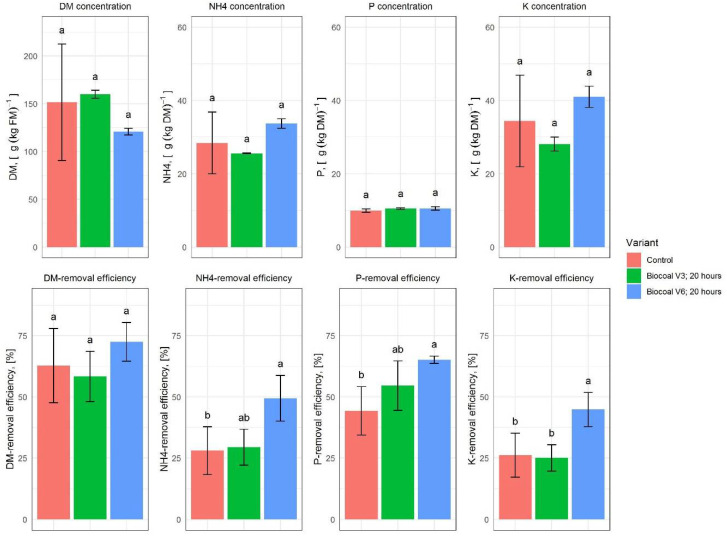
Comparison of the control variant (untreated digestate) with the two most promising additive variants and their best reaction times. Results were obtained in laboratory experiments. The upper diagrams show the concentrations. The lower diagrams show the removal efficiencies. Histograms are charted based on the mean values; error bars indicate the variability between the three replications. Lower case letters indicate significant differences according to the Tukey test.

After evaluating the results in Section 3.4, it can be concluded that the application of Biocoal V3 and Biocoal V6 for the tested reaction time of 20 h resulted in the highest removal efficiency. The comparison of the results of these two variants with the control from the laboratory conditions is shown in Figure 9.

The results represented revealed that pretreatment of digestate with Biocoal V6 over the 20 h reaction time resulted in significantly higher mean removal efficiencies for NH_4_, P, and K in the solid phase after separation compared to the control and the other variants studied. The application of Biocoal V6 resulted in a mean NH_4_-removal efficiency, which was 76.33% higher than for the control under laboratory conditions and 67.81% higher than after pretreatment with Biocoal V3. The mean P-removal efficiency after Biocoal V6 application was 47.15% higher than for the control and 19.37% higher than after application of Biocoal V3. The K-removal efficiency in the solid fraction after the pretreatment with Biocoal V6 was 71.06% higher than the control and 78.67% higher than after application of Biocoal V3.

### 3.6. Summary of Results and Further Needed Research

In this study, the effect of various biocoal-based additives on nutrient removal during the separation of digestate was investigated at laboratory scale and at a full-scale biogas plant. A separation methodology was developed for the laboratory-scale, which led to similar results compared to the full-scale separation experiments. To improve the nutrient removal efficiency and increase the nutrient content in the solid phase after separation, the digestate was pretreated with different biocoal additives. First, the biocoals used in this research were tested on their specific water uptake and other characteristics. Then, the effects of pyrolysis temperature and pretreatment of the wood before pyrolysis on the sorption capacity of the biocoal was investigated. The digestate pretreatment with the biocoals impregnated with MgCl_2_ for 20 h, synthesized at either 350 °C or 600 °C, resulted in higher nutrient recovery compared to the other tested variants. The application of Biocoal V6 for 20 h resulted in a significantly higher removal efficiency of NH_4_, P and K compared to the untreated digestate and other tested variants. The enhanced adsorption effect of Mg-modified biocoal produced from corn stalk at 450 °C on ammonium nitrogen and phosphate was confirmed in the study [44]. In [44], the aforementioned biocoal has been investigated on its agronomic effect in pot analyses, proving the nutrient slow-release ability and the promoting of plant growth.

Reducing the particle size of the biocoal by additional crushing in the mill could improve its sorption characteristics and lead to a higher nutrient removal efficiency. In addition to different reaction times, testing of higher temperatures (i.e., 40 °C) is also recommended and is currently being investigated in a follow-up research project.

## 4. Conclusions

A nutrient recovery in the solid fraction of the digestate has been investigated. A laboratory-scale digestate separation technology equivalent to full-scale pressure filtration has been developed. The nutrients recovery from the untreated digestate separated in the laboratory experiments, corresponded to the control variant. Pretreatment of the digestate with different additives before separation was tested in the laboratory conditions and the two most promising variants with the highest nutrient recovery in the solid fraction were identified: Biocoal from beech wood impregnated with MgCl_2_, synthesized at either 350 °C or 600 °C and applied over a reaction time of 20 h. After the application of biocoal impregnated with MgCl_2_ (600 °C, 20 h), the removal efficiency was increased by 76.33% for NH_4_, 47.15% for P and 71.06% for K compared to the control variant at the laboratory-scale.

## Figures and Tables

**Table 1:** Experimental design of the digestate pretreatment with additives. The check marks indicate the experiments performed. The added amounts corresponded to 1.2 kg of the digestate.

Variant	Additive, in g	Reaction Time					
		5 min	1 h	3 h	20 h	1 Week	2 Weeks
Biocoal V1, (350 °C)	6.05	✓	✓	✓	✓	✓	
Biocoal V2 (impregnated with CaCl_2_, 350 °C)	8.35				✓	✓	✓
Biocoal V3 (impregnated with MgCl_2_, 350 °C)	8.30	✓	✓	✓	✓	✓	✓
Biocoal V5 (impregnated with CaCl_2_, 600 °C)	8.30				✓	✓	✓
Biocoal V6 (impregnated with MgCl_2_, 600 °C)	5.79				✓	✓	✓
Commercial biocoal	9.99				✓	✓	✓
Commercial biocoal + MgCl_2_	9.99 + 1.78				✓	✓	✓

**Table 2:** Operating conditions of the research biogas plant “Unterer Lindenhof” and the properties of the digestate during the experimental period. OLR: organic leading rate; HRT: hydraulic retention time; FM: fresh matter; NA: not available.

Parameter	Reactor #1	Reactor #2	Secondary Reactor
OLR, in kg_oDM_∙(m³∙d)^−1^	3.42	3.26	2.24 *
			0.41 **
HRT, in d	61.50	58.00	32.40
Temperature (mean ± SD), in °C	44.00 ± 2.90	42.20 ± 3.20	52.50 ± 5.00
pH	NA		8.20 ± 0.41
DM, in %FM	NA		7.54 ± 0.84
oDM, g∙kg_DM_^−1^	NA		681.16 ± 15.23
NH_4_, g∙kg_DM_^−1^	NA		61.58 ± 3.46
P, g∙kg_DM_^−1^	NA		13.86± 0.32
K, g∙kg_DM_^−1^	NA		83.37 ± 5.35
Ca, g∙kg_DM_^−1^	NA		26.78 ± 1.34
Mg, g∙kg_DM_^−1^	NA		7.76 ± 0.39

* Feeding plus digestate from primary digesters. ** Only feeding.

**Table 3:** Elemental analysis of the biocoals in % of the dry weight.

Variant	C, in%	H, in%	N, in%
Biocoal V1 (350 °C)	64.32 ± 3.00	1.95 ± 0.49	0.26 ± 0.02
Biocoal V4 (600 °C)	77.56 ± 6.07	2.08 ± 0.48	0.25 ± 0.03

**Table 4:** Laboratory analyses of the biocoals.

Variant	Bulk Density (Mean ± SD), in kg∙m^−3^	Particle Size (Mean ± SD), in mm	DM, (Mean ± SD), in g∙kg_FM_^−1^	oDM, (Mean ± SD), in g∙kg_DM_^−1^	Ca, (Mean ± SD), in g∙kg_FM_^−1^	Mg, (Mean ± SD), in g∙kg_FM_^−1^
Biocoal V1, (350 °C)	176.55 ± 13.34	7.00 ± 3.00	1024.61 ± 2.72	969.97 ± 2.29	3.66 ± 0.58	1.53 ± 0.51
Biocoal V2 (impregnated with CaCl_2_, 350 °C)	261.43 ± 1.53	10.00 ± 5.00	1047.02 ± 2.93	549.71 ± 3.59	165.79 ± 8.73	0.52 ± 0.04
Biocoal V3 (impregnated with MgCl_2_, 350 °C)	243.55 ± 5.47	9.00 ± 3.00	1041.02 ± 0.98	811.11 ± 2.79	3.10 ± 0.19	101.16 ± 3.31
Biocoal V4, (600 °C)	158.19 ± 2.36	5.00 ± 2.00	1002.23 ± 0.92	1006.03 ± 98.63	7.95 ± 1.40	2.74 ± 0.79
Biocoal V5 (impregnated with CaCl_2_, 600 °C)	284.48 ± 0.61	7.00 ± 3.00	1030.99 ± 0.57	482.36 ± 11.06	166.11 ± 22.29	0.56 ± 0.01
Biocoal V6 (impregnated with MgCl_2_, 600 °C)	217.11 ± 1.44	7.00 ± 3.00	1033.45 ± 2.31	489.31 ± 3.41	3.67 ± 0.09	145.05 ± 4.68

## Data Availability

Not applicable.
